# Gastric Mucosal Biopsy Revealing Immunoglobulin G4 (IgG4)-Related Disease With Subtle Macroscopic Changes: A Case Report

**DOI:** 10.7759/cureus.103772

**Published:** 2026-02-17

**Authors:** Takeo Inaji, Tsuneaki Kenzaka, Hiroyuki Mori, Norimitsu Uza

**Affiliations:** 1 Department of Internal Medicine, Hyogo Prefectural Tamba Medical Center, Tamba, JPN; 2 Division of Community Medicine and Career Development, Kobe University Graduate School of Medicine, Kobe, JPN; 3 Division of Gastroenterology, Department of Internal Medicine, Kobe University Graduate School of Medicine, Kobe, JPN

**Keywords:** case report, endoscopic findings, gastric mucosal biopsy, gastrointestinal involvement, igg4-positive plasma cells, igg4-related disease

## Abstract

Immunoglobulin G4 (IgG4)-related disease affects multiple organs throughout the body, including the pancreas, bile ducts, lacrimal glands, salivary glands, lungs, kidneys, retroperitoneum, and lymph nodes; however, reports of gastrointestinal involvement are scarce. We report the case of a 40-year-old Japanese woman who presented with bilateral eyelid edema and enlargement of the submandibular and parotid glands. Upper gastrointestinal endoscopy was performed due to epigastric discomfort. Although the endoscopy revealed minimal mucosal abnormalities, the gastric mucosal biopsy demonstrated marked IgG4-positive plasma cell infiltration. These findings provided sufficient pathological evidence to establish a diagnosis of IgG4‑related disease. The patient was treated with steroid therapy, which was highly effective and resulted in rapid symptom improvement. This case highlights that in patients with gastrointestinal symptoms, a gastric mucosal biopsy is valuable for the definitive diagnosis of IgG4-related disease, even when gross endoscopic findings are limited.

## Introduction

Immunoglobulin G4 (IgG4)-related disease is a systemic chronic inflammatory disease characterized by elevated serum IgG4 levels and histological evidence of IgG4-positive plasma cell infiltration [1]. It can involve multiple organs, including the pancreas, bile ducts, lacrimal glands, salivary glands, lungs, kidneys, retroperitoneum, and lymph nodes [1]. However, reports of gastrointestinal tract involvement are rare [2]. Lesions have been reported throughout the digestive tract, including in the esophagus [3], stomach [4], duodenum [4], small intestine [5], and large intestine [6], and typically present as subepithelial tumors or ulcers [2,4]. We report a case of IgG4-related disease diagnosed by gastric mucosal biopsy, despite minimal endoscopic abnormalities.

## Case presentation

A 40-year-old Japanese woman presented with facial swelling. She first noticed swelling of her jaw 8 months before presentation, and developed swelling at the outer corners of her eyes three months before presentation. One month before the current visit, she consulted an otolaryngologist who noted bilateral eyelid edema, bilateral submandibular and parotid gland enlargement, and a serum IgG4 level of 3,668 mg/dL (standard value: 11-121 mg/dL). She was referred to our hospital for further evaluation. Her symptoms included dry eyes, dry mouth, headache, generalized fatigue, upper and lower eyelid edema, submandibular and parotid gland enlargement, and epigastric discomfort. She did not present with fever, photosensitivity, visual impairment, stomatitis, hearing loss, dizziness, dysphagia, morning stiffness, muscle weakness, limb numbness, rash, Raynaud's phenomenon, bloody stools, or constipation.

She had a history of uterine fibroids but no other significant medical history. She used etizolam 0.5 mg daily, as needed for insomnia, but took no other medications. Her height was 161.5 cm, weight was 78 kg, and vital signs were as follows: blood pressure, 152/108 mmHg; pulse, 84 beats/min; respiratory rate, 15 breaths/min; temperature, 36.6 °C; and oxygen saturation (SpO2), 99% (room air). Bilateral eyelid, parotid, and submandibular gland swelling were also noted. Superficial lymph nodes were not palpable.

Blood test results were as follows: white blood cells, 7,940/μL (neutrophils 78.0% and lymphocytes 12.5%); CRP, 0.61 mg/dL; IgG, 3,955 mg/dL; IgG4, 4,164 mg/dL; IgA, 171 mg/dL; IgM, 71 mg/dL; rheumatoid factor, 101 IU/mL; antinuclear antibody, <40-fold; anti-SS-A antibody, 0.5 U/mL; and anti-SS-B antibody, 0.6 U/mL (Table 1).

**Table 1:** Patient characteristics on admission

Parameter	Recorded value	Standard value
White blood cell count	7,940/µL	4500–7500/µL
Neutrophils	78.0%	42–74%
Lymphocytes	12.5%	18–50%
Hemoglobin	11.9 g/dL	11.3–15.2 g/dL
Platelet count	29.9 × 10^4^/µL	13–35 × 10^4^/µL
C-reactive protein	0.61 mg/L	≤0.60 mg/dL
Total protein	9.6 g/dL	6.9–8.4 g/dL
Albumin	3.6 g/dL	3.9–5.1 g/dL
Total bilirubin	0.4 mg/dL	0.2–1.2 mg/dL
Aspartate aminotransferase	15 U/L	11–30 U/L
Alanine aminotransferase	10 U/L	4–30 U/L
Lactase dehydrogenase	207 U/L	109–216 U/L
Creatine kinase	31 U/L	40–150 U/L
Blood urea nitrogen	9.8 mg/dL	8–20 mg/dL
Creatinine	0.73 mg/dL	0.63–1.03 mg/dL
Sodium	136 mEq/L	136–148 mEq/L
Potassium	4.2 mEq/L	3.6–5.0 mEq/L
Chloride	102 mEq/L	98-108 mEq/L
Glucose	95 mg/dL	70–109 mg/dL
IgG	3,955 mg/dL	870-1700 mg/dL
IgG4	4,164 mg/dL	11-121 mg/dL
IgA	171 mg/dL	110-410 mg/dL
IgM	71 mg/dL	46-260 mg/dL
Rheumatoid factor	101 IU/mL	<15 IU/mL
50% hemolytic complement activity	13 U/mL	25.0-48.0 U/mL
Complement C3	90 mg/dL	86-160 mg/dL
Complement C4	14.1 mg/dL	17-45 mg/dL
Anti-SS-A antibody	0.5 U/mL	<10.0 U/mL
Anti-SS-B antibody	0.6 U/mL	<10.0 U/mL
Antinuclear antibody	<40-fold	<40-fold
Anti-Sm antibody	1.0 U/mL	<10.0 U/mL
Anti-U1-RNP antibody	1.3 U/mL	<10.0 U/mL
Urinalysis		
pH	5.5	5.0-8.5
Protein	(-)	(-)
Occult blood	(-)	(-)
Urinary sediments		
Red blood cell	<1/HPF	<4/HPF
White blood cell	1-4/HPF	<4/HPF
Microbe	(1+)	(-)

**Figure 1:**
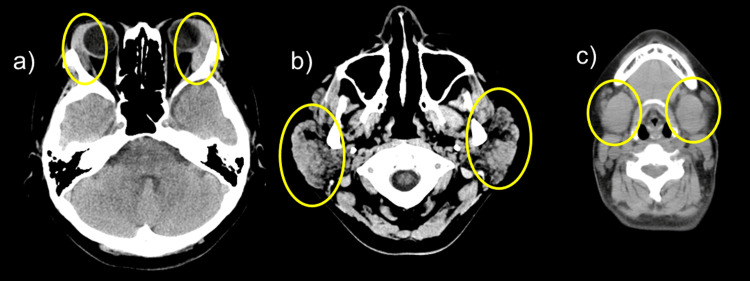
Head and neck CT scan without contrast a) Swelling of the bilateral lacrimal, b) parotid, and c) submandibular glands (yellow circles) CT, computed tomography

**Figure 2:**
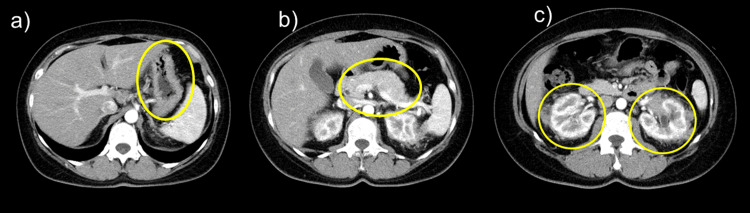
Contrast-enhanced CT of the chest and abdomen CT shows mild thickening of the gastric wall (a) and slight enlargement of the pancreas without a capsule-like rim (b). The kidneys exhibit mild contour irregularity with increased density of the surrounding fat and thickened fascia (c) (yellow circles). CT, computed tomography

Plain head and neck CT revealed swelling of the bilateral lacrimal, parotid, and submandibular glands (Figure 1). Contrast-enhanced chest and abdominal CT showed mild thickening of the gastric wall. The pancreas was slightly enlarged, and no capsular structures were observed. Both kidneys exhibited a slightly distorted shape, and the surrounding fatty tissue showed increased density and thickened fascia (Figure 2).

Minor salivary gland biopsy revealed marked infiltration of lymphocytes and plasma cells. Immunohistochemistry showed an IgG4/IgG ratio of ≥40%, with IgG4-positive plasma cells exceeding 10 per high-power field.

**Figure 3:**
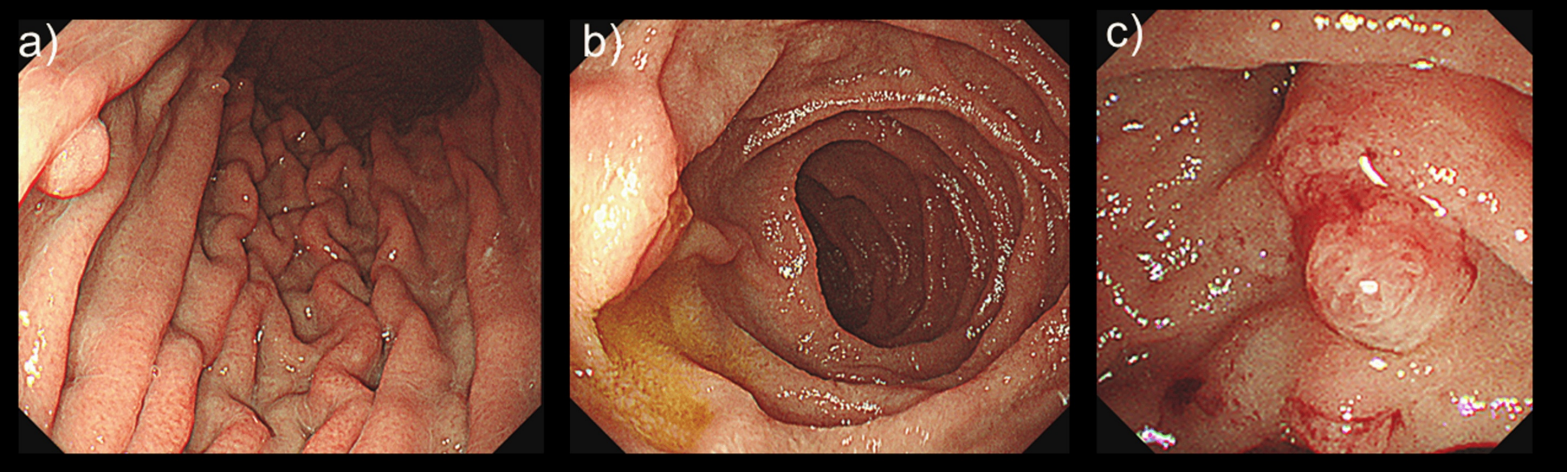
Upper gastrointestinal endoscopy image a) stomach, b) duodenum, c) duodenum Prominent gastric folds are observed from the upper to the lower body; however, the stomach is adequately distended with insufflation and no mucosal abnormalities are noted.

Upper gastrointestinal endoscopy was performed because of the epigastric discomfort. The stomach showed prominent folds from the upper to the lower body, but distension with insufflation was adequate, and no mucosal abnormalities were noted (Figure 3). The duodenal papilla showed no obvious enlargement on direct endoscopy, and no significant findings were observed on lateral endoscopy. Biopsies were obtained from all these sites.

**Figure 4:**
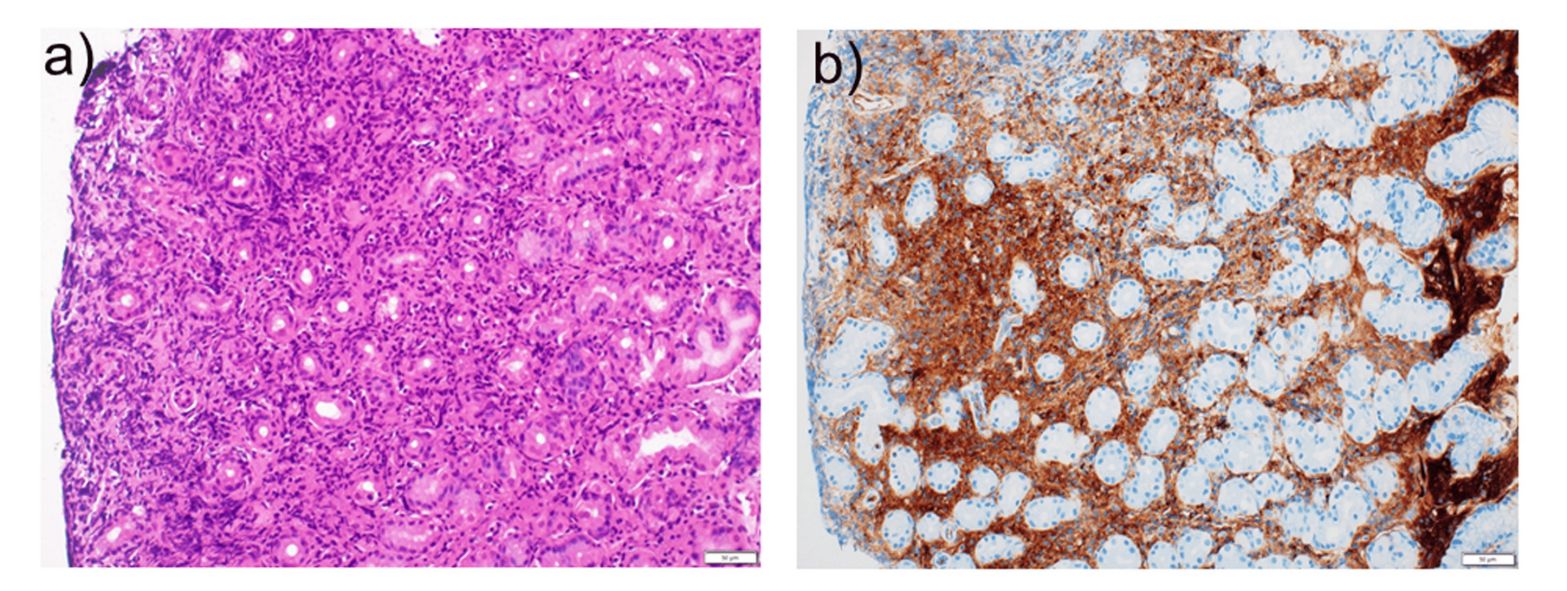
Histopathological examination of the gastric mucosa a) Hematoxylin and eosin staining (×20), b) IgG4 immunostaining (×20) Hematoxylin and eosin (H&E) staining revealing significant lymphocytic and plasma cell infiltration; IgG4 immunostaining revealing an IgG4/IgG ratio of ≥40%, with IgG4-positive plasma cells exceeding 10/HPF HPF, high-power field

The comprehensive diagnostic criteria for IgG4-related diseases [7] include clinical features, such as enlargement of multiple organs (lacrimal glands, parotid glands, submandibular glands, pancreas, and kidneys), serologically confirmed elevated IgG4 levels, and histologically demonstrated marked lymphocytic and plasma cell infiltration, with IgG4-positive plasma cell infiltration in the minor salivary glands and stomach/duodenum. In this case, gastric mucosal pathology findings revealed marked lymphocytic and plasma cell infiltration (Figure 4). Immunohistochemistry demonstrated an IgG4/IgG ratio of ≥40% and IgG4-positive plasma cells exceeding 10 per high-power field. These findings established a definitive diagnosis of IgG4-related disease.

Treatment was initiated with prednisolone 40 mg (0.6 mg/kg). Swelling of the eyelids, parotid glands, and bilateral submandibular glands improved rapidly. Epigastric discomfort also resolved, and elevated IgG4 levels gradually normalized. Prednisolone was tapered gradually. Twelve months after initiating treatment, the prednisolone dose was reduced to 10 mg/day, with no recurrences observed.

## Discussion

We report a case of IgG4-related gastrointestinal disease diagnosed using histology, which revealed marked IgG4-positive plasma cell infiltration meeting the diagnostic criteria [7], as the endoscopic findings were unremarkable.

Previous reports of IgG4-related gastrointestinal lesions have primarily described endoscopically visible lesions, including subepithelial tumors, ulcers, and erosions [2,4]. Although rare, several cases of IgG4-related gastrointestinal disease with no gross abnormalities have been reported; however, the biopsy findings were consistent with IgG4-related disease [8].

IgG4-related disease is a systemic fibroinflammatory condition characterized by tumefactive lesions, dense lymphoplasmacytic infiltration rich in IgG4-positive plasma cells, storiform fibrosis, and obliterative phlebitis [9]. Because serum IgG4 elevation lacks specificity, and imaging findings often overlap with malignancies or other inflammatory disorders, histopathological confirmation remains the gold standard for diagnosis [10,11]. The international consensus statement emphasizes that tissue biopsy is indispensable not only for confirming IgG4-related disease but also for excluding key differential diagnoses such as lymphoma, Sjögren’s syndrome, and malignancy [11].

In gastrointestinal involvement, the diagnostic challenge is even greater. Gastrointestinal lesions often present with subtle or nonspecific endoscopic findings, and the true prevalence of gastrointestinal IgG4-related disease may be underestimated due to limited biopsy sampling [4]. Several reports have highlighted that even macroscopically normal mucosa can harbor diagnostic IgG4-positive plasma cell infiltration, underscoring the importance of obtaining tissue whenever clinical suspicion exists [4].

Histopathological confirmation is essential for a definitive diagnosis of IgG4-related gastrointestinal disease. Minor salivary gland biopsy is minimally invasive but has limited sensitivity (approximately 50%) for detecting IgG4-related disease [12]. A major salivary gland biopsy may provide more reliable tissue sampling. However, submandibular gland excision carries the risk of mandibular nerve injury, while parotid gland biopsy carries risks of facial nerve injury and cosmetic concerns [13]. In IgG4-related pancreatic disease, the reported biopsy sensitivities were 47% for the pancreas, 47% (8/17) for the gastric mucosa, 36% for the liver, 0% for the bile duct, and 57% (4/7) for the duodenal papilla [14]. These findings highlight the heterogeneity of organ-specific diagnostic yields and underscore the need for strategic biopsy site selection.

Therefore, obtaining biopsies from multiple organs may improve diagnostic accuracy, particularly in cases where clinical presentation is atypical or when serological markers are inconclusive. The presented case suggests that, even when gross gastric mucosal findings are minimal, a biopsy may reveal pathological IgG4 positivity, aiding in diagnosis. This finding reinforces the growing recognition that gastrointestinal biopsies, though often overlooked, can serve as a valuable diagnostic adjunct in systemic IgG4-related disease.

## Conclusions

We report a case of IgG4-related disease diagnosed by gastric mucosal biopsy despite minimal gross endoscopic findings. This case underscores that even subtle or nonspecific gastrointestinal symptoms may warrant tissue sampling, as histopathological evaluation can reveal clinically significant IgG4-positive plasma cell infiltration. Early recognition through strategic biopsy selection may facilitate the timely initiation of steroid therapy and prevent disease progression.

Our findings highlight the diagnostic value of gastric mucosal biopsy in patients with suspected IgG4-related disease, even when endoscopic abnormalities are subtle or absent. Increased awareness of this diagnostic approach may improve the detection of gastrointestinal involvement and contribute to a more comprehensive evaluation of systemic IgG4-related disease.
